# *Nanos2* marks precursors of somatic lineages and is required for germline formation in the sea anemone *Nematostella vectensis*

**DOI:** 10.1126/sciadv.ado0424

**Published:** 2024-08-16

**Authors:** Andreas Denner, Julia Steger, Alexander Ries, Elizaveta Morozova-Link, Josefine Ritter, Franziska Haas, Alison G. Cole, Ulrich Technau

**Affiliations:** ^1^ Department of Neurosciences and Developmental Biology, Faculty of Life Sciences, University of Vienna, Djerassiplatz 1, 1030 Vienna, Austria.; ^2^ Research platform SINCEREST, University of Vienna, Djerassiplatz 1, 1030 Vienna, Austria.; ^3^ Max Perutz labs, University of Vienna, Dr. Bohrgasse 7, 1030 Vienna, Austria.

## Abstract

In animals, stem cell populations of varying potency facilitate regeneration and tissue homeostasis. Notably, germline stem cells in both vertebrates and invertebrates express highly conserved RNA binding proteins, such as *nanos*, *vasa*, and *piwi*. In highly regenerative animals, these genes are also expressed in somatic stem cells, which led to the proposal that they had an ancestral role in all stem cells. In cnidarians, multi- and pluripotent interstitial stem cells have only been identified in hydrozoans. Therefore, it is currently unclear if cnidarian stem cell systems share a common evolutionary origin. We, therefore, aimed to characterize conserved stem cell marker genes in the sea anemone *Nematostella vectensis*. Through transgenic reporter genes and single-cell transcriptomics, we identify cell populations expressing the germline-associated markers *piwi1* and *nanos2* in the soma and germline, and gene knockout shows that Nanos2 is indispensable for germline formation. This suggests that *nanos* and *piwi* genes have a conserved role in somatic and germline stem cells in cnidarians.

## Teaser

The differentiation of germline and soma in *Nematostella vectensis* involves the germline genes *nanos2* and *piwi1*.

## INTRODUCTION

Cnidarians are well known for their high regenerative potential, sexual and asexual reproduction, and the potential for extreme longevity with some species being even considered immortal (*1*–*6*). Other metazoans, which share these features, facilitate them through multi- or pluripotent stem cell populations like the archaeocytes of sponges or the neoblasts of planarians (*7*, *8*). However, it is currently unclear if the diverse stem cell systems observed in the animal kingdom are homologous.

The comparison of stem cells in bilaterians with those of basally branching animals, such as cnidarians, may shed light on their potential common ancestry. The freshwater polyp *Hydra* has four independent stem cell populations, the ectodermal and endodermal epithelial stem cells, and two lineage-related subpopulations of interstitial stem cells (ISCs): one that gives rise to gametes and one that gives rise to gland, mucous, nematocytes, and neuronal cells (*9*–*13*). Yet, ISCs have so far only been observed in hydrozoans, where they can be easily identified morphologically and by a high nucleus-to-cytoplasm ratio. For the rest of the cnidarian phylum, the source of new cells (i.e., their adult stem cells) for any tissue is unknown. While in several non-hydrozoans, “amoebocytes” have been described, their differentiation status and relationship to interstitial cells are unclear (*10*). The lack of clear evidence of ISCs outside of hydrozoans suggests that this cell type may be an innovation of this lineage rather than a synapomorphy of the Cnidaria as a whole (*10*). This would be somewhat unexpected given that most non-hydrozoan cnidarians have similar growth and regeneration capacities as hydrozoans. Hence, if there are no ISCs outside of hydrozoans, the question arises: Which cells can fulfill their function? Alternatively, they might have been simply overlooked by the so far classic histological methods used to address this question. Therefore, we aimed to elucidate the characteristics of the ancestral state for application to evolutionary questions and deduce common principles of cnidarian stem cells by using the anthozoan *Nematostella vectensis* as a model system.

Since in *Nematostella* no cell population similar to the hydrozoan ISCs has been described, we performed a molecular characterization of potential stem cells through the expression of stemness-associated marker genes (*14*). As an alternative way to the identification of stem cells by morphological features, we reasoned that conserved molecular markers of stem cells might point us to cell populations with stem cell properties. *Piwi*, *vasa*, and *nanos* were found to be expressed in multipotent and pluripotent stem cells of various metazoans (*10*, *15*, *16*). As members of the so-called germline multipotency program (GMP), these genes are often expressed in or even restricted to the germline of ecdysozoan and vertebrate animals, although they can also be expressed in multipotent stem and progenitor cells in echinoderms, lophotrochozoans, cnidarians, and poriferans (*17*–*19*). *Piwi* genes interact with Piwi-interacting RNAs (piRNAs), which guide them to repress transposons and protect the genome from damage (*20*). In a parallel study, it has also been shown that the GMP genes *piwi1*- and *vasa2*-expressing cells differentiate into *soxB*(*2*)^+^ neural progenitor cells in *Nematostella*, thereby marking a putatively multipotent precursor population (*21*). The *nanos* gene family codes for zinc finger–containing RNA binding proteins, with apoptosis and differentiation promoting mRNA targets, thereby inhibiting those processes (*22*, *23*). The activity of both gene families is especially conserved in the germline of vertebrates and insects, where they ensure genomic integrity and keep gametes in a stem cell–like state (*24*–*27*).

In *Nematostella*, we found three piwi genes (one of which appears to be a pseudogene) (*28*) and two *nanos* paralogs, *nanos1* and *nanos2*. While *nanos1* marks the neuronal progenitor cells (*14*), *nanos2* expression studies implied a role in germline formation, similar to that of *vasa1*, *vasa2*, and *PL10* (*29*). Furthermore, *nanos* gene expression can be detected in the ISCs of *Hydractinia* and *Hydra* (*30*, *31*). Together, these expression studies of *nanos* suggested a role in the germline, multipotent stem cells and neuronal progenitor cells.

In this study, we used single-cell transcriptome data to identify a population of *piwi1-* and *nanos2*-positive cells. We generated transgenic reporter lines using *piwi1* and *nanos2* promoters and showed that both cell populations give rise to somatic cell types of the neuroglandular lineages. A *nanos2* knockout (KO) mutant reveals a conserved crucial role for *nanos* in the formation of the germline.

## RESULTS

### *Piwi1* and *Nanos2* are expressed in a broad range of somatic cell types and gametes

**Fig. 1.**
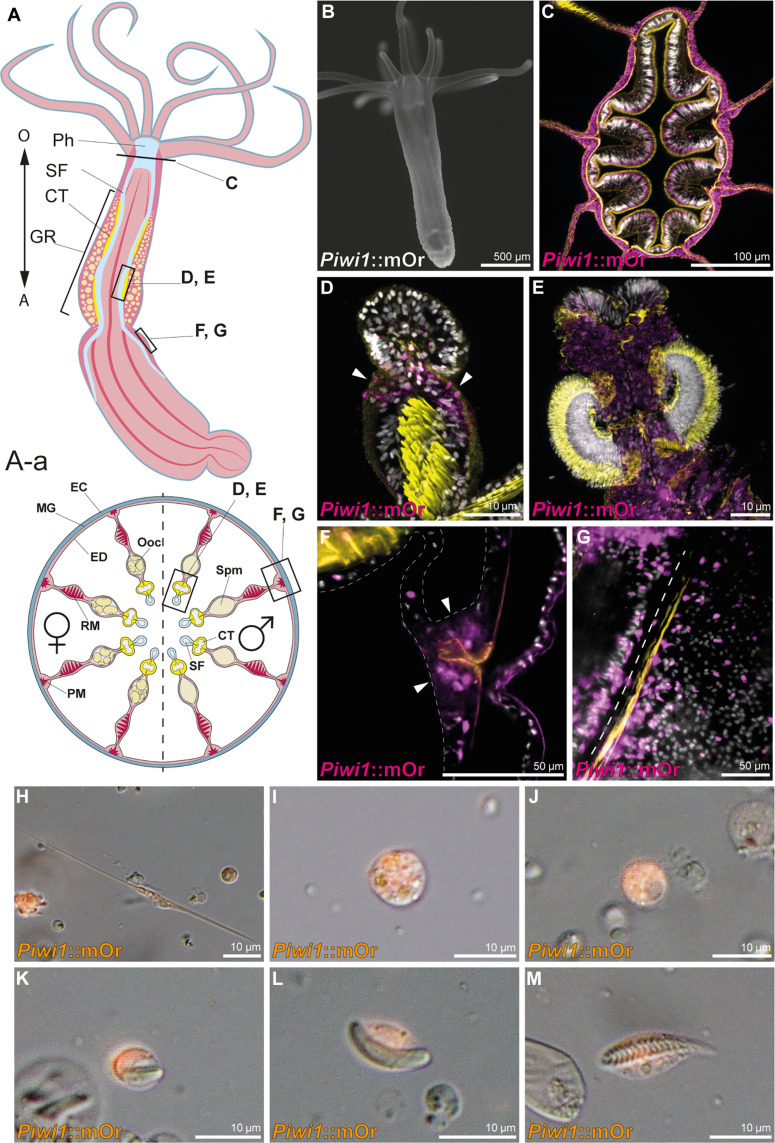
*Piwi1* reporter expression in somatic cell types. (**A**) Whole-body and transversal section schematic indicating tissues and location of *piwi1*::mOr-expressing cells. (A) O, oral pole; A, aboral pole; Ph, pharynx; SF, septal filament; CT, ciliated tract; GR, gonadal region. (A-a) EC, ectoderm; MG, mesoglea; ED, endomesoderm; PM, parietal muscle; RM, retractor muscle; Ooc, oocytes; Spm, spermaries. (**B**) Juvenile *piwi1*::mOr polyp. mOr is displayed as white. (**C**) Section through a pharynx. (**D**) Juvenile mesentery. Arrows mark *piwi1*^+^ cells accumulating at the gonad primordium. (**E**) Ciliated tract of an adult mesentery. (**F**) *Piwi1*^+^ cells concentrated at the mesentery base (arrowheads), surrounding the parietal muscle (orange). (**G**) Optical section along the mesentery base showing *piwi1*^+^ cells along the parietal muscle; the dotted line indicates the mesentery base. (**H** to **M**) *Piwi1*::mOr-positive cell types. (H) Neuron. (I) Gland cell. (J) Oocyte. (K) Immature nematocyte. (L) Mature nematocyte. (M) Spirocyte. For all fluorescent pictures, 4′,6-diamidino-2-phenylindole (DAPI) is displayed as white, and phalloidin is displayed as yellow.

*Piwi1* has been previously shown to be expressed broadly during early development (*28*); however, expression at later stages has not yet been well described and the fate of the *piwi1*-expressing cells is still unclear. To visualize *piwi1* expression in vivo, we generated a transgenic reporter line expressing mOrange2 (mOr) under the control of the *piwi1* promoter. The fluorescent reporter recapitulates the mRNA expression observed by in situ hybridization (ISH) (fig. S1) (*18*) shifting from a ubiquitous expression in the gastrula stage to mainly being restricted to endomesodermal tissues during polyp metamorphosis. A low level of reporter expression in the ectodermal body wall and tentacle epithelia is, however, retained from the polyp stage onward (Fig. 1, B and F). In the adult animal, the endodermal tissue surrounding the pharynx and the gonadal region of the mesentery and ciliated tract are also mOr-positive (Fig. 1C). In juvenile animals, reporter expression is more restricted to few cells residing between the distal tip of the retractor muscle and the ciliated tract, the region where the gonad will form during sexual maturation (Fig. 1D, arrowheads). In the adult mesenteries, the endomesodermal expression extends to all epithelia surrounding the gonads and the reticulate tract (Fig. 1E). Notably, large numbers of small single cells displaying mOr reporter expression are found all along the mesenteries, but especially concentrated around the parietal muscle (Fig. 1, F and G).

To identify cell types expressing *piwi1*::mOr, we dissociated adult animals and surveyed single cells. In these cell spreads, we find the *piwi1*-driven fluorescent reporter protein in all oocytes (Fig. 1J) and in descendants of the neuroglandular lineage: neurons (25% mOr^+^; Fig. 1H), gland cells (41% mOr^+^; Fig. 1I), and cnidocytes (19% mOr^+^; Fig. 1, K to M). In summary, *piwi1::*mOr is expressed in oocytes, spermatogonia, putative germline stem cells, a fraction of cells of the neuroglandular lineage, and at low levels in the ectodermal body wall epithelia as well as the endomesodermal epithelia surrounding the gonadal region and the pharynx. This situation is similar to hydrozoan cnidarians, where *piwi* expression has been detected in somatic and germline cell types (*32*–*34*).

**Fig. 2.**
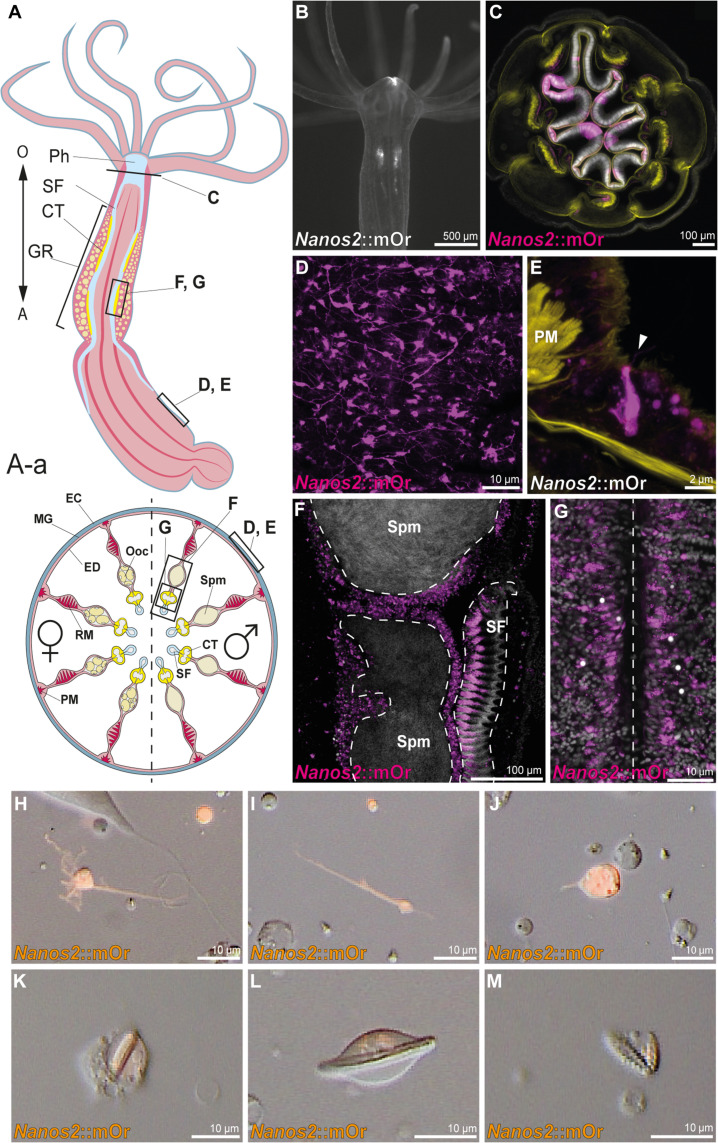
*Nanos2* reporter expression in somatic cell types. (**A**) Whole-body and transversal section schematic indicating tissues and location of *nanos2*::mOr-expressing cells. (A) O, oral pole; A, aboral pole; Ph, pharynx; SF, septal filament; CT, ciliated tract; GR, gonadal region. (A-a) EC, ectoderm; MG, mesoglea; ED, endomesoderm; PM, parietal muscle; RM, retractor muscle; Ooc, oocytes; Spm, spermaries. (**B**) Juvenile *nanos2*::mOr polyp. mOr is displayed as white. (**C**) Section through a pharynx. (**D**) Neuronal net in the body wall of an adult polyp expressing *nanos2* reporter. (**E**) *nanos2* reporter–positive sensory neuron in the endomesoderm next to the parietal muscle. Sensory cilium is indicated by an arrowhead. (**F**) Vertical section through a male gonad and ciliated tract. The epithelia surrounding the spermaries and the bordering septal filament express *nanos2*::mOr. (**G**) Optical section along the mesentery base showing *nanos2*::mOr positive cells along the parietal muscle; the dotted line indicates the mesentery base. (**H** to **M**) *Nanos2*::mOr–positive cell types. (H and I) Neurons. (J) Gland cell. (K) Immature nematocyte. (L) Mature nematocyte. (M) Spirocyte. For all fluorescent pictures, DAPI is displayed as white, and phalloidin is displayed as yellow.

We next wished to study the lineage derivatives of *nanos*-expressing cells. A phylogenetic analysis shows that the *nanos* gene has duplicated in the common ancestor of all cnidarians and most cnidarians have retained both paralogs (fig. S2). *Nanos1* has recently been found to mark the neural precursor cells in *Nematostella* (*14*). To monitor the expression and fate of *nanos2*-expressing cells in *Nematostella*, we generated a transgenic reporter line using the upstream and downstream cis-regulatory elements expressing mOrange2. The fluorophore expression during developmental stages recapitulates the pattern retrieved from ISH, displaying ubiquitous expression during early development and an up-regulation in the gastrodermal primary mesenteries of the primary polyp (fig. S1). In juvenile transgenic animals, *nanos2* reporter protein is expressed around the mouth (Fig. 2B), similar to *Hydra* (*31*), in the ciliated tracts of the primary mesenteries below the pharynx, as well as in single cells distributed throughout the whole animal in both germ layers (Fig. 2B). The ectodermal layer displays a dense net of neurons (Fig. 2D), expressing the mOr fluorophore at low levels with undifferentiated cells rarely dispersed throughout the layer, whereas in the endomesodermal layer, sensory neurons, gland cells, and undifferentiated cells are concentrated along the parietal muscle (Fig. 2G). The pharynx shows patchy expression in the ectodermal tissue [which, according to recent profiling, has an endodermal identity (*35*)] with parts of the innermost folds and the outward folds bordering the inner layer expressing the reporter gene (Fig. 2C). Similar to the *piwi1* transgenic line, *nanos2*::mOr is expressed in the epithelia surrounding the gonad; however *nanos2*::mOr additionally marks the side of the ciliated tract bordering the gonadal regions, corresponding to the reticulate tract (Fig. 2F). In the single-cell spreads, we can again detect *nanos2* reporter protein expression in all neuroglandular cell types (Fig. 2, H to M). In contrast to *piwi1*, almost all neurons were *nanos2*::mOr*^+^* (87%), whereas nematocytes (19%) and gland/gastrodermis cells (32%) showed a comparable ratio of mOr reporter expression. However, other than neurons which are almost all *nanos2*::mOr^+^, only a fraction of neuroglandular cell types show low levels of residual fluorophore, and most are not expressing mOr in both the *piwi1* and *nanos2* transgenic animals. Therefore, we conclude that both genes are most likely expressed in the precursor population, from which the neuroglandular cell types differentiate and carry on residual mOr reporter protein.

Together, both *nanos2* and *piwi1* reporter lines show a very similar expression pattern in terms of cell types. The *nanos2* promoter drives fluorophore expression in all descendants of the neuroglandular lineage and the gonad epithelia. However, in contrast to *piwi1*::mOr, *nanos2::*mOr is not expressed in oocytes. It also marks the epithelia around the mouth, in the pharynx, and the putative gametogenic region of the reticulate tract in the septal filament (*36*).

**Fig. 3.**
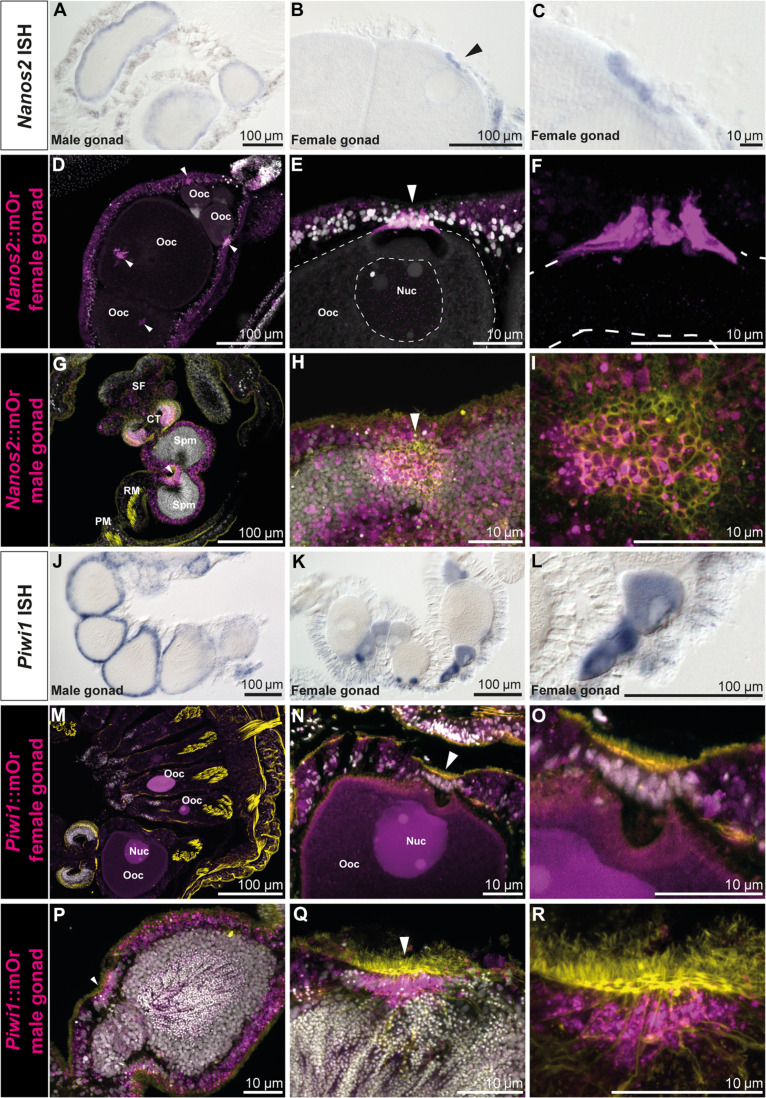
*Nanos2* and *piwi1* mRNA and reporter expression in gametes and the gonadal region. (**A**) *Nanos2* expression in male mesenteries. (**B**) *Nanos2* expression in female mesenteries. *Nanos2^+^* trophocytes are marked by an arrow. (**C**) Detailed picture of (B). (**D** to **I**) *Nanos2*::mOr expression in adult gonads. (D) Female gonad showing reporter-expressing trophonemata located closely to the germinal vesicle of each oocyte (arrowheads). (E) *Nanos2*::mOr-expressing trophonema (arrowhead). Nuc, nucleus of the oocyte. (F) Detailed picture of (E) (DAPI not displayed). (G) Male gonad, with mOr expressing in the gastrodermis around the spermaries and the bordering tissue of the ciliated tract. The ciliated plug is indicated by an arrowhead. (H) Detailed picture of a ciliated plug (arrowhead) in the gastrodermis surrounding the sperm chambers. Cells composing the plug can be identified by increased mOr expression and phalloidin incorporation (yellow). (I) Detailed picture of (H) (DAPI not displayed). (**J**) *Piwi1*::mOr expression in male mesenteries. (**K**) *Piwi1*::mOr expression in female mesenteries. (**L**) Detailed picture of the mOr^+^ maturing oocytes in (K). (**M** to **R**) *Piwi1*::mOr expression in adult gonads. (M) Female gonad. (N) Oocyte with the mOr^−^ trophonema indicated by an arrowhead. (O) Detailed picture of (N). (P) Male gonad expressing *piwi1*::mOr in the endomesoderm surrounding the sperm chambers. The ciliated plug is indicated by an arrowhead. (Q) A ciliated plug (arrowhead) in the gastrodermis surrounding the sperm chambers. Cells composing the plug can be identified by increased mOr expression and phalloidin incorporation (yellow). (R) Detailed picture of (Q) (DAPI not displayed). For all fluorescent pictures, mOr is displayed as magenta, DAPI is displayed as white, and phalloidin is displayed as yellow.

As both *nanos* and *piwi* genes are conserved germline factors, we studied their expression in detail in the gonadal region. Both genes show the same expression pattern in males where they are expressed in the outer perimeter of the spermaries (Fig. 3, A and J). In females, however, *nanos2* marks the trophocytes (accessory cells), which are situated close to the germinal vesicle of each oocyte (Fig. 3, B to F). These cells form the trophonemata, a cnidarian-specific ring-shaped structure consisting of multiple trophocytes, which are in close contact with the surface of the oocyte and thought to provide nutrients to the growing egg (Fig. 3, D to F, arrowheads) (*37*, *38*). A plug of ciliated cells, which express both *nanos2* and *piwi1*, is apparent at each sperm cyst. This structure has been described previously and has been speculated to be homologous to the trophonemata (Fig. 3, H, arrowhead, and I) (*39*).

*Piwi1* is expressed in the oocytes themselves, where the reporter concentration is highest in smaller oocytes and fading in larger ones (Fig. 3, K and L). However, *piwi1* expression is lacking from the trophonemata (Fig. 3, N, arrowhead, and O) but can also be detected in the ciliated plugs of the spermaries (Fig. 3, P to R, arrowheads). To our knowledge, this plug has not been described in detail in *Nematostella* and we can only speculate about its function. It is apparent that the size of the nuclei in the sperm cysts decreases from the outer margins toward the inside, suggesting that sperm differentiation proceeds from the perimeter inward. These small nuclei, however, seem to flow toward the ciliated plugs, which indicates that this might be the place where sperm is released into the body column during spawning (Fig. 3, G, H, P, and Q).

Through the expression patterns obtained by ISH and the reporter gene expression in transgenic animals, we conclude that both *piwi1* and *nanos2* reporter proteins are carried over to differentiated somatic cell types of the neuroglandular lineage and the germline. The mOr reporter of both genes is detected only in a few cells of each of the somatic cell types. This suggests that the fluorophore in these fully differentiated cells was carried over from a common neural progenitor cell (NPC). However, *piwi1* reporter expression is much stronger in the germline, whereas *nanos2*-positive cells are much more frequent in the descendants of somatic lineages. Also, both markers are expressed in epithelial cell populations, where *piwi1* is very broadly expressed in the body wall ectoderm at a low level, while *nanos2* is restricted to regions of the pharynx and ciliated tract, with a higher expression level overall. To assess the implications on differentiated cell populations, we therefore focused on surveying the *nanos2*-expressing population.

### *Nanos2^+^* cells are a cycling population

**Fig. 4.**
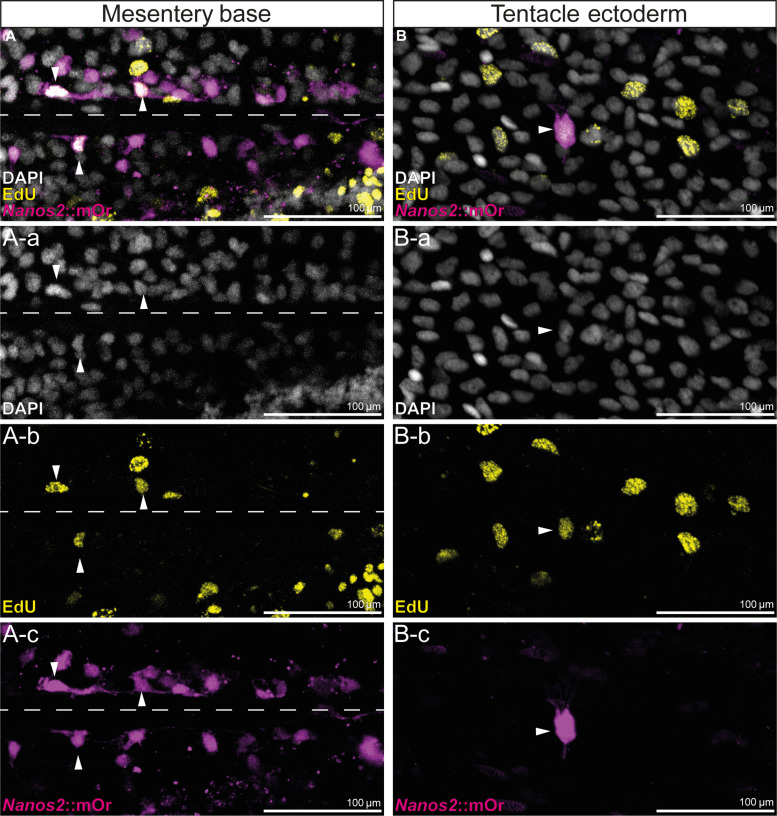
EdU incorporation of *nanos2*::mOr-expressing cells. (**A**) A-a to A-c: Fluorescent pictures of the endoderm at the base of the mesentery. The attachment site of the mesentery is indicated by a dotted line. mOr/EdU^+^ cells are indicated by arrowheads. (**B**) B-a to B-c: Fluorescent pictures of the tentacle ectoderm. mOr/EdU^+^ cell is indicated by an arrowhead.

To assess whether the *nanos2*::mOr-expressing cells comprise an actively cycling population, we labeled transgenic animals for 24 hours with EdU. After this period, a fraction of the mOr^+^ cells in each body region and tissue were marked by EdU (Fig. 4). Since the transgenic line retains mOr reporter expression in derivatives of *nanos2*-expressing cells, we suspect that the EdU-negative *nanos2*::mOr^+^ cells are composed of terminally differentiated cell types such as neurons and gland cells as well as possibly slow cycling quiescent stem cells, whereas the fast cycling cells may be immediate precursor populations of the neuroglandular or germline cells.

To determine this fraction of actively cycling *nanos2*-expressing cells, we wished to carry out double staining of *nanos2* mRNA and EdU pulse labeling. However, the cellular resolution of the ISH of *nanos2* in adults is very limited also because the density of small *nanos2-*expressing cells is so high in many regions of the body. Therefore, we subjected the transgenic animals to an EdU pulse of 5 hours, dissociated them afterward, and counted the numbers of EdU-positive and -negative cells as well as *nanos2*::mOr-positive and -negative cells (table S1). We found that *nanos2*::mOr cells comprise a substantial fraction of all cells (23%), of which 11.8% were EdU^+^ under these conditions. This shows that at least a subpopulation of *nanos2*-positive cells is proliferative. Together with our finding that the mOr reporter protein is expressed in somatic lineages, this is consistent with the possibility that *nanos2*-expressing cells have properties of multipotent stem cells or progenitors. However, we also note that among the *nanos2*::mOr-negative cells, a similar fraction (13.3%) is also labeled by EdU. The nuclei of these *nanos2*-negative/EdU^+^ cells are relatively large and round, raising the possibility that these might be epithelial cells. While the nature of these cycling cells remains elusive, this shows that *nanos2*-expressing cells are not the only cycling cells in an adult polyp.

**Fig. 5.**
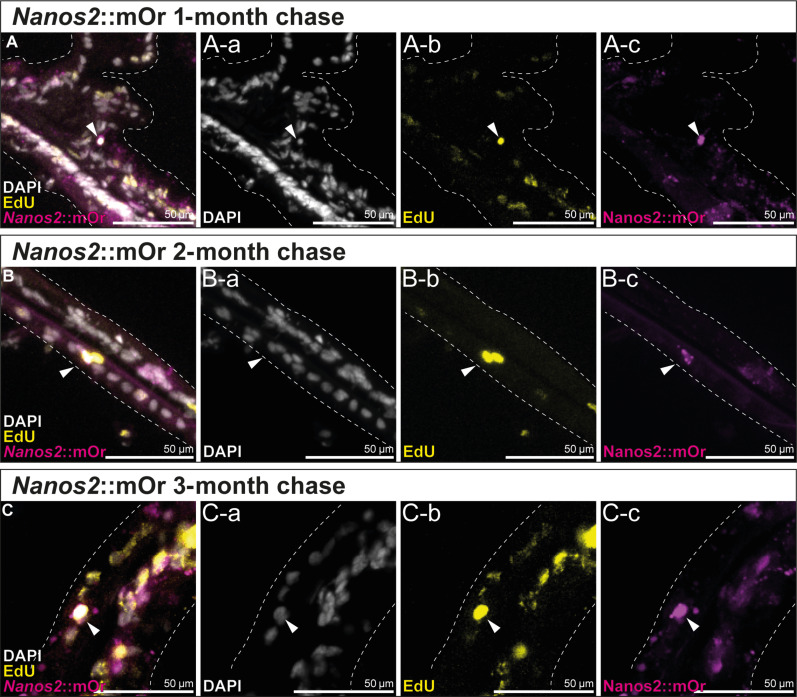
Long-term EdU label-retention assay shows that *nanos2*::mOr marks slow cycling, quiescent cells over 3 months. (**A** to **C**) EdU signal is retained in a few mOr^+^ cells in the body wall and mesentery epithelia over 3 months, marking a subpopulation of slowly cycling cells that still proliferate, indicated by EdU^+^ cell doublets (B). (A) One-month label retention. (A-a) DAPI only. (A-b) EdU only. (A-c) mOr only. (B) Two-month label retention. (B-a) DAPI only. (B-b) EdU only. (B-c) mOr only. (C) Three-month label retention. (C-a) DAPI only. (C-b) EdU only. (C-c) mOr only.

In other organisms including mammals, some adult stem cells are mitotically quiescent for long times and rarely divide (*40*, *41*). These can only be detected by a label retention assay, where an EdU pulse is followed by long periods of no label. Under such conditions, rapidly cycling progenitors would rapidly dilute out the incorporated EdU label to the progeny cells, while quiescent or rarely dividing stem cells would retain the label even after an extended time. To test whether *Nematostella* might have slow or quiescent stem cells, we incubated the *nanos2*::mOr (Fig. 5) and *piwi1*::mOr transgenic polyps (fig. S3) for 1 week in EdU, followed by an extensive chase with *Nematostella* medium (NM) and fixed the animals after several time points up to 3 months after the labeling. Figure 5 shows that in cryosections of the polyps, we found several EdU^+^/*nanos2*:mOr^+^ cells 1 to 3 months after the pulse, demonstrating the existence of quiescent or rarely dividing stem cells. Since the label could be also retained by differentiated, long-lived cells, e.g., neurons, it is important to note that the morphology of these cells were small round cells, intercalated between the epithelial cells throughout the animal, but often in the vicinity of the mesentery base, suggesting that these are nondifferentiated cells, similar to interstitial cells in hydrozoans. Thus, the detection of rarely dividing *nanos2*^+^ cells explains, at least in part, the relatively low fraction of pulse-labeled cells.

### *Nanos2* mutants fail to form primordial germ cells and gametes

To test the function of *nanos2* in cellular differentiation, we generated a KO mutant using CRISPR-Cas9 (*42*), introducing a 1–base pair (bp) insertion at the 5′ end of the predicted Nanos-specific zinc finger sequence (fig. S4). This results in an early stop codon, which renders the protein unable to form a ternary complex with binding partners such as Pumilio or bind to mRNAs on its own (*43*, *44*). After crossing the heterozygous mutant F1 parent generation, we observed no increased mortality or developmental defects in the offspring, and homozygous mutant animals were only identifiable through sequencing of the *nanos2* locus. We raised the homozygous mutant polyps to adulthood and tried to spawn them. While wild-type (WT) polyps start to spawn after about 4 to 5 months after fertilization, even after 1 year, none of the mutants spawned any gametes, neither oocytes nor sperm, suggesting that the *nanos2* mutants are sterile.

**Fig. 6.**
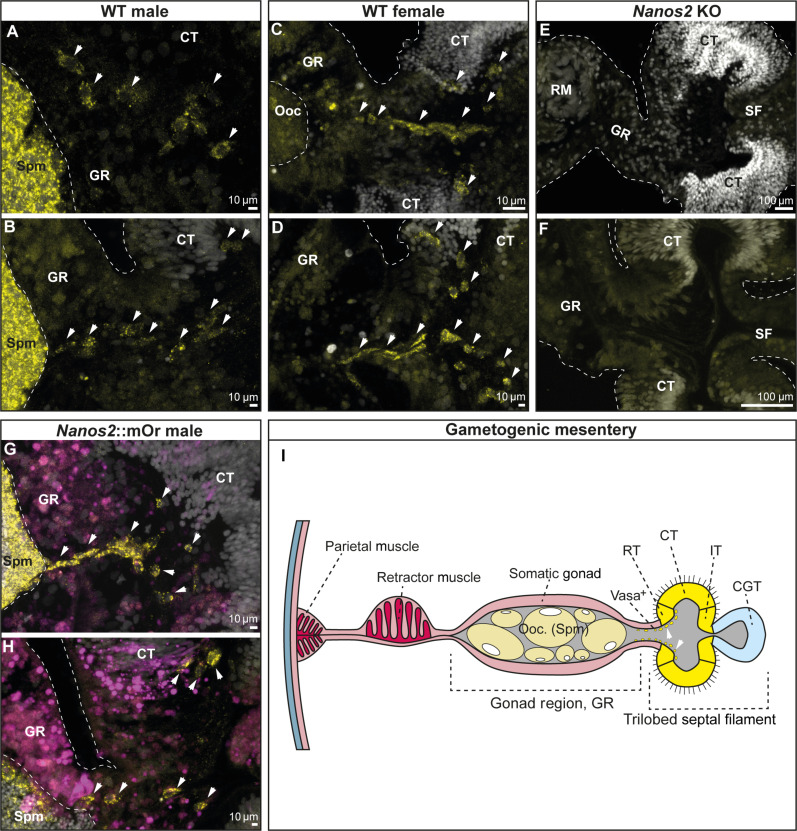
Vasa2 immunostaining of PGCs confirms the absence of germline cells in *nanos2*^−/−^ mutants. (**A** to **H**) Transversal sections of the gonadal region and trilobed septal filament of WT male (A and B), WT female (C and D), *Nanos2* KO (E and F), and *Nanos2*::mOr animals [(G and H) mOr fluorophore is depicted in magenta] stained with DAPI (white) and Vasa2 antibody (yellow). Vasa2^+^ cells have been indicated by white arrows. Note that no Vasa2^+^ cells nor mature oocytes or spermatocytes could be detected in *Nanos2* mutants, explaining the sterility of the animals after 12 months. If not otherwise indicated, scale bars correspond to 10 μm. (**I**) Schematic of a gametogenic female mesentery with a trilobed septal filament. Vas^+^, Vasa2-positive putative primordial germ cells; ret. t., reticulate tract; cil. t., ciliated tract; int. t., intermediate tract; cni.-gla. t., cnido-glandular tract.

To further validate this, we carried out staining for the Vasa2 protein. Vasa has been established as a marker for the germline in *Nematostella*, *Hydra*, and several other animals (*17*, *28*). In *Nematostella*, the Vasa2 protein has been shown to mark putative germline stem cells during development, spermatogonia, oocytes, and single cells in the epithelium surrounding the gonads (*28*). In line with that, in WT animals, Vasa2^+^ primordial germ cells (PGCs) are visible between the septal filament and the gonad, closely associated with the reticulate tract, a region of the ciliated septal filament most closely bordering the gonad (Fig. 6, C to F and K). *nanos2*^−/−^ animals on the other hand display somatic gonads lacking oocytes or sperm and devoid of Vasa2^+^ cells (Fig. 6, G and H). This result is coherent with the observed sterility of adult mutants.

### Nanos2 is required for germline formation but dispensable in somatic cell type lineages

**Fig. 7.**
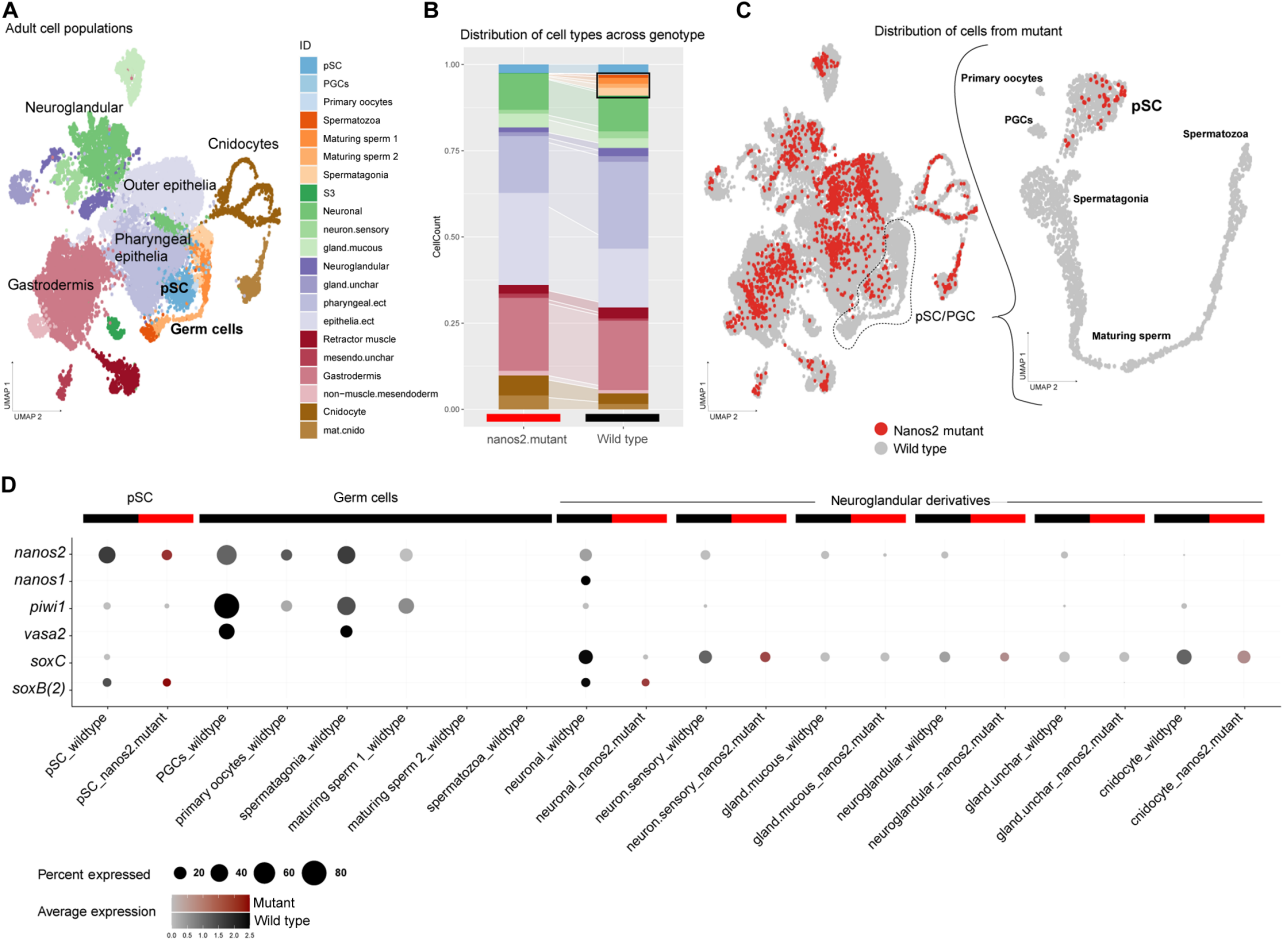
Single-cell RNA-seq analysis of the *nanos2*^−/−^ mutant reveals the absence of primordial germ cells and gametes. (**A**) Single-cell transcriptomic map of an individual *nanos2*^−/−^ mutant juvenile polyp merged with WT adult tissue libraries. (**B**) Library contribution to the map in (A) illustrates a similar contribution of the mutant to all principal cell populations with the exception of germ cells and uncharacterized cell type S3 (black outline). (**C**) Distribution of mutant cells (red) across the entire dataset (gray), and the cluster of putative stem cells (pSCs) and germ cells identifies the absence of germ cells and differentiated gametes in *nanos2*^−/−^ mutants. (**D**) Expression dot plot of pSCs, germ cells, and neuroglandular derivative cell populations. Detection of gene expression in at least 5% of the cell population is illustrated. Germ cell marker expression (*nanos2*, *piwi1*, and *vasa2*) shows all three genes are coexpressed and elevated within the WT germ cell populations, as well as detected in both mutant and WT pSCs. *nanos2* is also broadly detected across all populations in the WT samples and is partially coexpressed with markers of early neuroglandular progenitors *nanos1*, *soxC*, *and soxB*(*2*), whose expression is unaffected in the mutant.

The characterization of our *nanos2::mOr* and *piwi1*::mOr transgenic reporter lines revealed fluorophore expression in a variety of somatic cell types in both cell layers (see Figs. 1 to 3). To assess the consequences of the *nanos2*^−/−^ mutation on the cell type composition of the animal, we carried out single-cell RNA sequencing (scRNA-seq) of an individual *nanos2*^−/−^ mutant 2-month-old juvenile polyp. We combined the *nanos2*^−/−^ mutant cell transcriptomes with the available WT adult tissue catalog (*14*, *45*), newly mapped to the updated genome and transcriptome models (*46*). We processed the combined dataset and assigned cluster identities according to Cole *et al*. (*47*). Cells that express markers of neuroglandular precursors [*soxB*(*2*), *neurogenin*, *soxC*, etc.] cluster together with early germ cells (expressing *PROF-2*, *Boule-like*, etc.; table S3).To investigate the distribution of the mutant-derived cells within the putative stem cell (pSC) and primary germ cell population, we reanalyzed this cluster separately (Fig. 7C, right). We then imported cell state identities from this subset to the full integrated dataset for visualization (Fig. 7A). The distribution of cells from the *nanos2*^−/−^ mutant indicates that the mutant sample exhibits no differences in the overall somatic cell type diversity (Fig. 7, B and C), further supporting the observation that the mutant is vital and shows no apparent deficiencies of somatic tissues.

In the WT, we detect elevated levels of *nanos2*, *piwi1*, and *vasa2* expression in the pSC/PGC clusters (Fig. 7D). Of note, these genes show partial overlap with the early regulators of the neuroglandular lineages *nanos1*, *soxC*, and *soxB2a* [also known as *soxB*(*2*)]. PGCs originate at the boundary of the ectodermal and mesodermal cell layer in the pharynx of primary and juvenile polyps, from where they migrate into the gonad primordia (*36*). Within this subset of the single-cell data of the WT polyp, we can clearly identify the maturing spermatozoa, primary germ cells, and a cluster of primary oocytes in addition to the pSC population (Fig. 7C). We find no cells from the *nanos2*^−/−^ mutant sample contributing to the PGCs, although this sample does contribute to the pSC precursor population (Fig. 7C). Neither male nor female gametes could be detected in *nanos2*^−/−^ mutants. This suggests that *nanos2* is required for the formation of the PGCs in both sexes and the subsequent differentiation of gametes at an early stage of development. Together with the morphological observations described above, we conclude that *nanos2* is required for germline formation and gamete differentiation in *Nematostella*.

We note that in the single-cell transcriptomes of the *nanos2^−/−^* mutants, the derivatives of the neuroglandular lineages are still present (Fig. 7, A to C), although neuroglandular derivatives are found in the *nanos2* transgenic line, suggesting a lineage relationship (Fig. 2). We previously described *nanos1* marking the neuronal progenitor population, overlapping with *soxC*, which acts as the most upstream determinant of several somatic lineages, such as cnidocytes, neurons, and gland cells (*14*). This is in line with the observation that the mutants are phenotypically normal and can feed and move like WT animals. Long-term observations of our animals did not indicate defects in the homeostatic renewal of neuroglandular cell types or a negative impact on the survivability of *nanos2*^−/−^ mutants. We also could not detect any notable difference in the regeneration capacity of the mutants compared to WT animals (fig. S5). Therefore, while *nanos2* is expressed in NPCs, it is unlikely that *nanos2* is essential for the differentiation of the neuroglandular lineages into terminal cell types. However, we cannot rule out that the lack of a phenotype in the neuroglandular lineages in the *nanos2^−/−^* mutant might be due to genetic compensation, for instance, by *nanos1* (*48*). In line with this, *nanos2* and *nanos1* also show a partially overlapping expression (Fig. 7C).

**Fig. 8.**
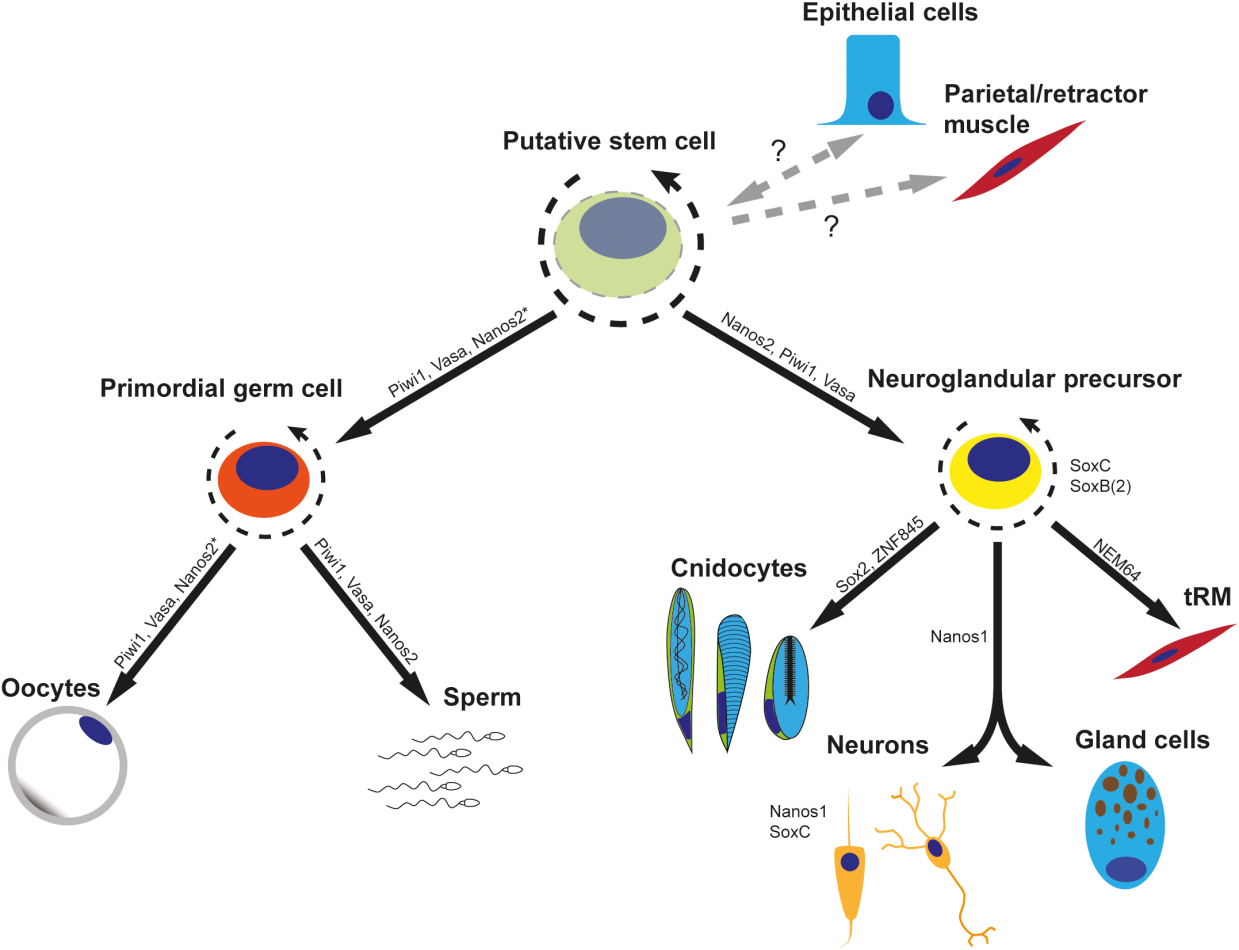
Working model of *Nematostella* stem cell differentiation. The presence of a putative multipotent stem cell able to differentiate into germline cells and all cell types of the neuroglandular lineage is implicated by the lineage tracing experiments using stable reporter proteins. The relationship of this cell population with differentiated epithelial cells and retractor/parietal muscle cells is currently uncertain (indicated by dashed arrows and question marks). The expression of nanos2* leading to the germ cell lineage indicates that the gene is expressed in spermatogonia but not expressed in the oogonia; however, *nanos2* is indispensable for primordial germ cell formation and remains expressed in gonad-adjacent somatic cell types such as the trophocytes. Gene expression is depicted according to this work and Steger *et al*. (*14*).

The coexpression of *nanos2* and *piwi1* in the neural progenitor population and multiple derivatives, together with expression patterns of the fluorophore detected in our transgenic lines, indicates a role of *nanos2* and *piwi1* beyond the specification of PGCs. However, slight differences in gene function might be inferred by the single-cell expression data. *Nanos2* appears to feature a much broader expression profile than *piwi1* and is particularly up-regulated in the pSC population of both WT and mutant. On the other hand, *piwi1* and *vasa2* are highly expressed in the gametes rather than the pSCs. The lack of germline in the *nanos2* mutant animals suggests that *nanos2* might facilitate the differentiation of pSCs toward *piwi1*/*vasa2*^+^ gametes, although *nanos2* is no longer expressed in the gametes themselves. As *piwi1* and *vasa2* are also expressed in the pSC population, further studies are needed to determine whether *nanos2* acts epistatically or if multiple of these germline factors are needed for germline formation. Together, our data suggest that *nanos2* and *piwi1* define early precursors that differentiate into most cell types of *Nematostella*, with the possible exception of epithelial cells. Therefore, we consider *nanos2-* and *piwi1*-positive cells as a population of pSCs that give rise to both PGCs and neural progenitor cells, reminiscent of the hydrozoan ISC populations (Fig. 8) (*49*).

## DISCUSSION

Cnidarians have remarkable tissue plasticity and regenerative capacity. They can grow and shrink repeatedly yet maintain a homeostatic ratio of all cell types at all times. However, many cell types, such as neurons, cnidocytes, and gland cells, are terminally differentiated. Gametes can also be considered a differentiated cell type. Therefore, they must be replenished from a pluripotent or multipotent stem cell pool, with the ability to form gametes as well as somatic cell types (*50*). In *Hydra*, ISCs have been shown to act as multipotent stem cells for all cells except epithelial cells (*51*). In the marine hydrozoan *Hydractinia*, interstitial cells have been shown to also give rise to epithelial cells; thus, they are considered pluripotent cells (*52*, *53*), similar to neoblasts in planarians. Yet, ISCs have so far only been identified in hydrozoans, and not in any of the other cnidarian classes, despite the fact that they have very similar properties. Therefore, we used conserved molecular stem cell marker genes to molecularly identify pSCs in the sea anemone *Nematostella*. This includes the *piwi* and *nanos* gene families, which have been implicated in the germline of many bilaterians (*43*, *54*–*63*). Piwi binds to piRNAs, which are silencing transposons, and hence is thought to contribute to the genome integrity of the germ cells (*27*). However, an involvement in somatic cells has also been demonstrated. For instance, in planarians, *piwi* was shown to be essential for the pluripotent stem cells, the neoblasts, which give rise to all other cell types and hence are crucial for the maintenance of the whole body (*64*–*66*). Similarly, in *Nematostella*, the *piwi1*::mOr reporter protein marks terminally differentiated cell types of the neuroglandular lineage. In addition, the *piwi1*::mOr transgenic line displays low-level expression in the ectoderm and gastrodermis. We could also detect mRNA transcripts of *piwi1* and *nanos2* in cells of these epithelia in scRNA-seq, but we were unable to detect them by ISH. The low-level expression of these genes might hint toward the existence of epithelial cells as separate stem cell lineages that replenish the epithelia of the polyp, similar to those of *Hydra*. This would be in line with the existence of a cycling cell population, which is negative for *nanos2*::mOr and *piwi1*::mOr.

Nanos, an RNA binding zinc finger protein implicated in translational repression (*67*), has also been implicated not only in the germline of many bilaterians but also in somatic stem cells of some highly regenerative animals, e.g., in neoblasts in planarians (*68*), archeocytes in sponges (*69*), and ISCs in *Hydra* (*31*). In *Hydra*, a transgenic reporter line under the control of the *nanos1* promoter showed that *nanos1*-positive stem cells give rise to cnidocytes and neurons (*70*). However, while the function of *piwi* and *nanos* has been shown in planarians, in *Hydra*, there are only expression studies available. Moreover, ISCs have only been demonstrated in hydrozoans, not in other cnidarians. Thus, the nature of possible multipotent stem cells remains enigmatic at this point. The expression of two *nanos* paralogs, *nanos1* and *nanos2* (common to all cnidarians), and two *piwi* genes, *piwi1* and *piwi2*, has been reported before (*28*, *29*). However, the cellular resolution of ISH did not permit visualization of individual cells in the adult. Moreover, neither their lineage relationship with other cell types nor their function is known. In the sea anemone *N. vectensis*, scRNA-seq and transgenic lines showed that *nanos1* is expressed in neuronal precursor cells and gives rise to neuronal cells (*14*). By contrast, scRNA-seq suggested that *piwi1* and *nanos2* are expressed in a broad spectrum of undifferentiated and differentiating cell types. In this study, we have generated transgenic lines of the *piwi1* and *nanos2* genes in the sea anemone *N. vectensis.* Both reporter lines mimic the mRNA expression derived by ISH and show similar, but not identical, patterns: *Piwi1* is expressed broadly at a low level in the ectodermal layer but is strongly up-regulated in many small round cells in the inner (endomesodermal) cell layer and in the early stages of germ cells (oogonia and spermatogonia). *Nanos2* and *piwi1* transgene expression is found in numerous very small cells located between epithelial cells of both layers, spread all over the whole body. The reporter gene expressions in both transgenic lines thereby reflect the scRNA-seq data of *nanos2* and *piwi1* marking precursor populations of the neuroglandular lineage. Since the fluorophore also transmits into differentiated somatic cell types, like neurons and gland cells, we assume that the *nanos2*^+^ cells give rise to differentiated cells of the neuroglandular lineage. In addition, at least a fraction of the *nanos2*^+^ cells are proliferating and the label-retention assay over 3 months demonstrates that some are very rarely dividing cells. Similarly, the *piwi1* transgenic line also harbors slow cycling label retention cells. These features are essential criteria for a multipotent stem cell population similar to the ISCs in *Hydra*. A transgenic *vasa2* line also showed somatic cell type derivatives such as neurons (*21*). Costaining of the *nanos2*::mOr line with Vasa2 antibodies (ABs) showed partial overlap between Vasa2-positive cells (presumably PGCs) and *nanos2*^+^ cells. These data strongly suggest the role of *piwi1*, *nanos2*, and *vasa2* in a population of multipotent stem cells.

However, our *nanos2^−/−^* mutant did not show any detectable effect on the neuroglandular lineage, suggesting that *nanos2* is not essential for the formation of this somatic lineage from *nanos2^+^* precursor cells. Yet, we cannot rule out that *nanos1*, which is expressed in the proneuronal progenitor cells (*14*), compensates for the loss of *nanos2* in the neuroglandular lineages. Future work will have to address this possibility. As no functional data are available for *piwi1* and *vasa2*, we do not know whether these genes are essential for neuroglandular lineages and whether the identified single cells are truly somatic stem cells.

Regardless of the role of *nanos2* in the somatic cell lineages, our *nanos2* KO mutant clearly shows that it is indispensable for the generation of PGCs and hence for the formation of male and female gametes. As a result, *nanos2* mutants are sterile. A sterility phenotype has been also observed for *nanos* KOs in the mouse (*57*) and *Drosophila* (*71*). In *Drosophila*, *Caenorhabditis elegans*, and mice, *nanos* is necessary for germ stem cell self-renewal and it prevents precocious differentiation (*72*–*74*). Since we cannot detect PGCs in the *nanos2* mutant in *Nematostella*, we assume that this function is conserved between bilaterians and cnidarians and dates back at least to the common ancestor about 600 million years ago. Notably, it has recently been proposed that the *piwi1*^+^ and Vasa2^+^ cell population is the source of gametes as well as SoxB(*2*)^+^ neurons based on lineage analyses of transgenic lines (*21*). Since *nanos2*^+^ cells and derivatives comprise an even larger cell population with overlapping Vasa2^+^ cells, our data support this claim and confirm that the Vasa2^+^ cell population residing in the septal filament is the source of gametes and neuronal cells in *Nematostella*.

In summary, our results highlight the broad use of germline multipotency genes in somatic and gametic cell types in evolutionary early-branching animals. We confirm the role of *nanos* as an ancestral key determinant of germline establishment in the earliest branching metazoan and likely a crucial regulator of somatic cell types as well.

## MATERIALS AND METHODS

### *Nematostella* culture

Polyps of *N. vectensis* were cultured in 1.6% artificial sea water (NM) and fed daily with *Artemia nauplii*. The animals were kept at 18°C in the dark, while spawning was induced by exposure to light and increased temperature (*75*). After fertilization, egg packages were treated with 3% cysteine in NM to remove the egg jelly. Larvae were kept at 21°C until further usage or until they reached the juvenile stage.

### Generation of single-cell suspensions

Animals were collected in precoated Eppendorf tubes and washed multiple times in NM. Cell dissociations for juvenile and adult *Nematostella* polyps were performed using papain [3.75 mg or 37.5 U/ml in phosphate-buffered saline (PBS), Sigma-Aldrich, P4762] and collagenase (1.25 mg or 1000 U/ml in PBS, Sigma-Aldrich, C9407) solution in a 1:1 mix, with dithiothreitol added to a final concentration of 3.5 mM. Whole juvenile animals and pieces of adult animals were dissociated in 1 ml of the collagenase/papain dissociation solution, overnight at room temperature. The tissue was then carefully pipetted with a cut pipette tip to dissociate into single cells. The state of the dissociation was regularly checked using samples of the solution under a light microscope. If clumps were no longer visible, then the cells were pelleted in a precooled centrifuge for 3 min at 0.4*g*. A washing step with 1% bovine serum albumin (BSA) in PBS was performed to stop dissociation after which the pelleted cells were resuspended in 1% BSA/PBS on ice. Cell viability and concentration were assayed using a Cellometer X2 (Nexcelom). Only cell suspensions with a minimum viability of 80% were used for single-cell sequencing.

### Single-cell imaging

For single-cell imaging, animals were dissociated as described above. The cell solution was then applied to Superfrost slides (Thermo Fisher Scientific) on which the desired imaging area was previously marked with a hydrophobic barrier pen (Thermo Fisher Scientific). The liquid was then taken off using a micropipette and replaced by 4% paraformaldehyde (PFA) in PBS for fixation. After 2 min, the fixative was taken off the slides, and the adhered cells were washed three times for 2 min each in PBS. For light microscopy, PBS was then replaced with VECTASHIELD (Vector Laboratories), and a cover slip was applied before imaging.

### Single-cell RNA sequencing

The single-cell suspension was loaded into the 10X Genomics platform for encapsulation. The sequencing library was prepared according to the standard 10X protocol and sequenced according to the manufacturer’s recommendations. Raw sequence data were mapped to the v.2 chromosome genome assembly and accompanying Nv2 gene models (*46*) using the 10X genomics Cell Ranger pipeline, without secondary analysis and force recovery of 10,000 cells per library.

### Single-cell transcriptomic analysis

Count matrices for WT adult libraries and the newly generated mutant sample were imported into R and processed with the R package Seurat v.4 (*76*). Data were merged and normalized with Seurat’s default “LogNormalize” function. The 500 most variable genes from each sample were identified with the “FindVariableGenes” function with default parameters. The combined gene set was scaled with the “ScaleData” function binned by sample origin (“split.by” function). Informative principal components (PCs) were chosen as those with SD > 2 and used as input for the clustering analysis. Dimensional reduction was performed with Uniform Manifold Approximation and Projection using the same PCs as in the clustering. For the sPC/PGC subset, the top 2000 variable genes were calculated, scaled across the entire dataset binned by sample, and then processed further as described above. An R script for generating the analysis and figures is available on our GitHub page (https://github.com/technau/Nanos2 and https://zenodo.org/doi/10.5281/zenodo.10911440).

### Generation of transgenic animals

The *nanos2* and *piwi1* transgenic lines were generated as described previously in (*77*). Epigenetic signatures were used to identify putative promoter and enhancer sequences which were then cloned and used to drive the expression of mOrange2 on a p-CRII-TOPO backbone (*78*). The plasmids were injected into fertilized eggs at a concentration of 25 ng/μl together with I-Sce1 meganuclease. F0 mosaic transgenic animals were then crossed to WTs to generate F1 heterozygous transgenics.

### RNA probe synthesis

For the generation of ISH RNA probes, RNA was extracted from mixed embryonic stages using TRIzol (Thermo Fisher Scientific) after the manufacturer’s protocol. cDNA was then synthesized using the SuperScript III reverse transcriptase (Thermo Fisher Scientific) in conjunction with random hexamer and oligo(dT) primers. Primers encompassing the probe sequences were designed using Primer3Plus (www.bioinformatics.nl/cgi-bin/primer3plus/primer3plus.cgi) and Primer Stats (www.bioinformatics.org/sms2/pcr_primer_stats.html). The probe sequences were then amplified by polymerase chain reaction (PCR). The PCR products were then run on an agarose gel to confirm the correct product sizes and purified using a PCR purification kit (Analytik Jena). The cloned sequences were then inserted into the pJet1.2 Vector (Thermo Fisher Scientific), which was then transformed into chemically competent Top 10 *Escherichia coli* bacteria (Invitrogen). The plasmids were then harvested via miniprep (Analytik Jena), and the desired probe sequences were confirmed using Sanger sequencing (Microsynth). Linearized probe templates were generated by PCR using a T7 or Sp6 promoter–containing primer. Probes were then synthesized using the T7 or Sp6 MEGAscript Kit (Invitrogen) and digoxigenin (DIG) RNA Labeling Mix (Roche) (*79*). The DIG-labeled RNA probes were stored in a dilution of 50 ng/μl in a 1:1 mix of formamide and deionized water at −20°C.

### In situ hybridization

For embryonic stages, ISH was performed on whole-mount 24-hour gastrula, 2- and 4-day planula larvae, and 8-day-old primary polyps. The animals were fixed in 4% PFA/PBS for 1 hour at room temperature, washed three times in PBS with 0.1% Tween 20 (PTW) for 5 min each, and then transferred into methanol in which they were stored at −20°C.

ISH was performed after a previously published protocol (*80*). Minor changes were introduced in the RNA probe detection step: Anti-Digoxigenin-AP (anti-DIG/AP) Fab fragments (Roche) were used in a dilution of 1:4000 in 0.5% blocking reagent (Roche) in 1× maleic acid buffer. After incubation overnight at 4°C, the whole mounts were washed in PTW 10 times for 10 min, rinsed twice with alkaline phosphatase buffer (AP buffer), and stained with nitro blue tetrazolium–bromochloroindolyl phosphate (NBT/BCIP) dissolved in AP buffer. The stained animals were then washed several times in PTW and infiltrated in 86% glycerol. ISH images were taken using a Nikon 80i compound microscope equipped with a Nikon DS-Fi1 camera.

### Adult tissue ISH

In preparation for ISH, adult polyps were relaxed using 7.5% MgCl_2_ which was also introduced into the body cavity through the mouth opening using a glass capillary. The animals were then fixed overnight in 4% formaldehyde in PTW on a rotator at 4°C. Afterward, the polyps were dissected into small pieces of ~5 × 2 mm and washed in 100% methanol. The methanol was regularly replaced until no orange pigment was washed out anymore. Until use, the tissue pieces were stored in methanol at −20°C.

ISH on adult tissues was performed as described previously in (*28*). In short, PBT (1× PBS with 0.2% Triton X-100) was used instead of PTW in all washing steps. A bleaching step was introduced after rehydration, consisting of an incubation in 0.5% H_2_O_2_/5% formamide/0.5× SSC in H_2_O for 5 min. Triethanolamine (TEA) washes were all performed with 1% TEA in PBT. An adapted hybe buffer was used for the prehybridization and hybridization steps, additionally containing 10% dextran sulfate (Sigma-Aldrich) and 1% blocking reagent (Roche). Prehybridization was extended to overnight, and the probe concentration was raised to 0.75 ng/μl. In between the 2× SSC and 0.2× SSC washes, a ribonuclease T1 incubation (1 U/μl in PBS) for 40 min was introduced to reduce background staining. Last, the anti-DIG/AP AB concentration was raised to 1:2000.

### Generation of *nanos2* knock-out mutant animals

Guide RNAs for CRIPR-mediated mutagenesis (*42*) were designed using the Chop-Chop online tool [http://chopchop.cbu.uib.no/ (*81*)] targeting the 3’ end of the Nanos-specific zinc finger RNA binding domain. The guide RNAs were ordered at IDT-DNA and injected together with Cas9 protein (500 ng/μl). Successful mutagenesis was determined by melting curve analysis of a PCR product spanning ~120 bp around the target site. Mosaic mutant animals were then crossed to WT polyps to generate F1 heterozygous mutant families. These were then cross-bred to retrieve F2 homozygous KO mutants. Mutant animals used in this study have a 497insG mutation in the *nanos2* gene, resulting in a frameshift and premature stop codon.

### Vasa2 immunostaining

For anti-Vasa2 staining, a previously published mouse anti-Vasa2 AB was used (*28*); however, the immunofluorescence protocol was modified. Adult polyps were starved for 24 hours and then relaxed using 7.5% MgCl_2_. They were then transferred into an ice-cold fixative consisting of 3.7% formaldehyde/0.4% glyoxal/0.1% methanol/0.2% Triton X-100 in 1× PBS. The fixative was also introduced into the mouth opening using a glass capillary. After 10 min, the animals were dissected into ~5 × 2 mm pieces in the fixative, and fixation was carried out for an additional 1 hour at 4°C on a rotator. After fixation, three quick washing steps in PBT were performed, followed by three permeabilization washes in PBT for 20 min each. The tissue pieces were then transferred into 100% methanol and washed at 4°C with regular replacement of methanol until it remained clear and no pigment dissolved anymore. Afterward, a 7-min wash in ice-cold 100% acetone was performed, followed by two washes in PBT. The tissue was then blocked for at least 2 hours at RT in a blocking buffer containing 1% BSA/5% sheep serum/20% dimethyl sulfoxide (DMSO)/0.2% Triton X-100 in 1× PBS. In parallel, the primary anti-Vasa2 AB was preabsorbed in AB blocking solution with 1% BSA/5% sheep serum/0.1% DMSO/0.2% Triton X-100 in 1× PBS at a concentration of 1:100. The samples were then incubated in an AB blocking buffer containing the AB overnight at 4°C. The samples were then washed 10 times for 10 min each in PBT to remove the primary AB. The secondary AB (goat anti-mouse 488, Sigma-Aldrich) was then preabsorbed in an AB blocking buffer and the samples were blocked in the same solution, before being incubated in the secondary AB-containing solution overnight at 4°C. Another 10 washing steps for 10 min in PBT were performed and the tissue pieces were then gelatin-sectioned and stained with 4′,6-diamidino-2-phenylindole (DAPI; 5 μg/ml). The sections were subsequently mounted onto slides and imaged using a confocal microscope (Leica Sp8).

### EdU staining

Transgenic animals were incubated in 20 μM EdU solution in 2% DMSO in NM for 5 hours for single-cell dissociations and for 24 hours when used for gelatin sections. For single-cell spreads, the animals were then dissociated using the collagenase/papain protocol and adhered to Superfrost slides as described above. After fixation, a permeabilization step using 0.5% Triton X-100 in PBS was performed for 30 min, followed by three washing steps in PBS and the EdU detection reaction as per the manufacturer’s protocol for 2 min on the slides.

For additional immunostaining, the cells were washed two times in PBT and subsequently incubated in a blocking solution consisting of 1% BSA and 20% sheep serum in PBT for 2 hours in a sealed humid chamber. The primary AB was also blocked for 2 hours and then slides were incubated with the AB solution overnight. To wash off the primary AB, five washing steps with PBT were performed. The slides and secondary AB were then again incubated in a blocking solution for 2 hours and the secondary AB was hybridized overnight. The secondary AB was once again removed through five washing steps in PBT. Before mounting, the cells were rinsed with PBS which was subsequently replaced by VECTASHIELD for imaging.

For gelatin sectioning, the animals were washed several times with NM after the EdU incubation and relaxed using 7.5% MgCl_2_ in NM, diluted 1:10. They were then fixed for 16 hours in 4% PFA in PBS at 4°C. The animals were then cut into smaller pieces of ~5 × 2 mm and washed for several hours in 100% methanol, regularly replacing the methanol until no more pigment was washed out. The tissue pieces were then rehydrated in PBS and put into 10% gelatin in PBS to infiltrate for 1 to 2 hours. The gelatin was then poured into molds, which were hardened at 4°C and refixed overnight in 4% PFA at 4°C. The gelatin plugs containing the tissue pieces were then rinsed in PBS again and 50-μm sections were prepared using a vibratome (Leica). The sections were then stored in PBS until they were incubated in DAPI and phalloidin and subsequently mounted.

### Animal welfare and ethics

*N. vectensis* is a simple, non-endangered invertebrate and does not require ethics approval.
