# Supplementary material for: Nanos2 marks precursors of somatic lineages and is required for germline formation in the sea anemone Nematostella vectensis
Source: Sci Adv. 2024 Aug 16;10(33):eado0424. doi: 10.1126/sciadv.ado0424 (PMC11328910; doi:10.1126/sciadv.ado0424)
Supplement: Supplementary file 1 — Figs. S1 to S5 Legends tables S1 to S3 [file sciadv.ado0424_sm.pdf]

Science Advances
AAAS

Supplementary Materials for

Nanos2 marks precursors of somatic lineages and is required for germline formation in the sea anemone *Nematostella vectensis*

Andreas Denner *et al.*

Corresponding author: Ulrich Technau, ulrich.technau@univie.ac.at

Sci. Adv. **10**, eado0424 (2024)
DOI: 10.1126/sciadv.ado0424

The PDF file includes:

Figs. S1 to S5
Legends tables S1 to S3

Other Supplementary Material for this manuscript includes the following:

Tables S1 to S3

Supplementary Materials

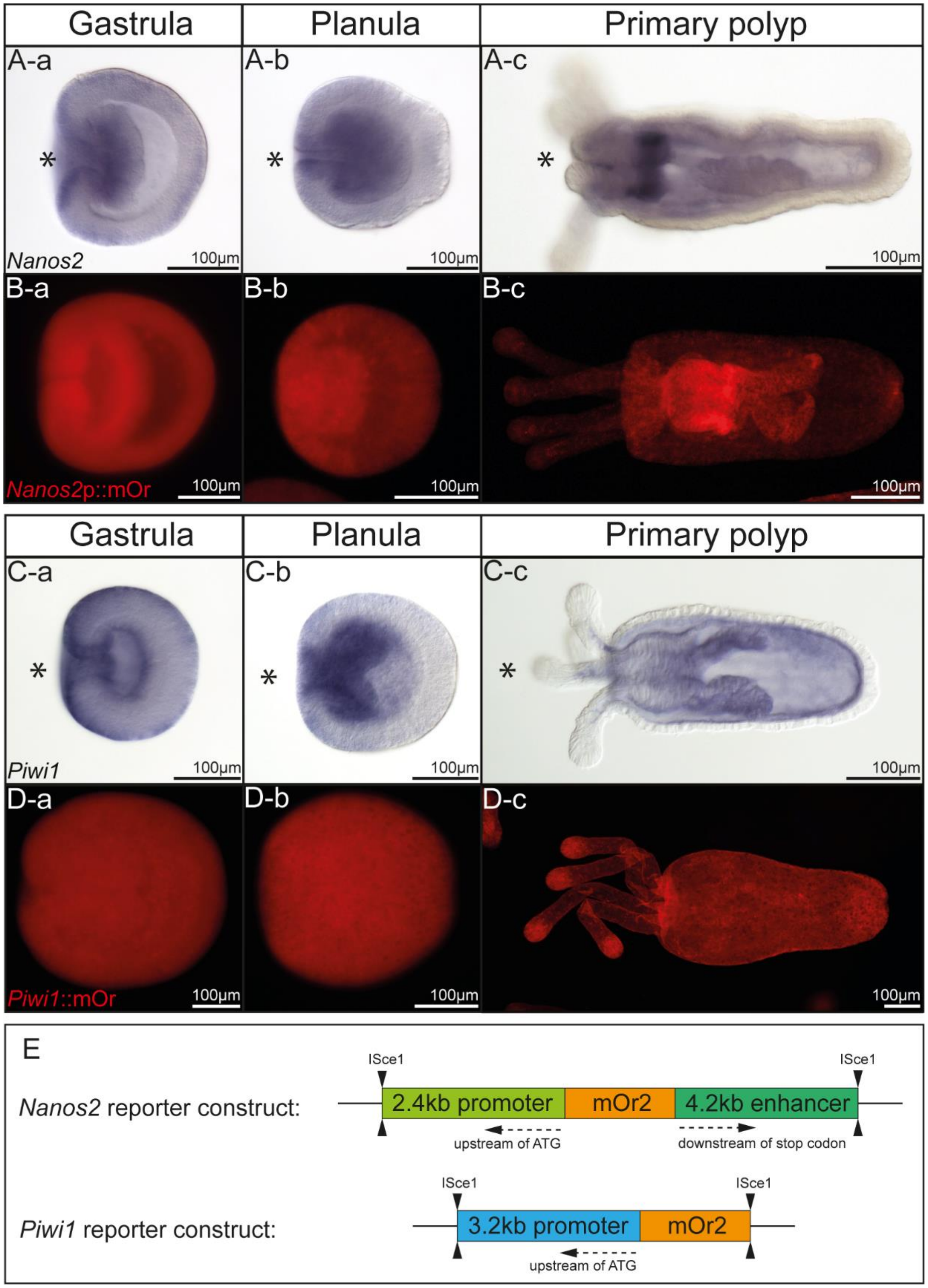

S1 Fig: *nanos2* and *piwi1* expression in developmental stages. a: 24h gastrula stage, b: 3d planula stage, c: 7d primary polyp; A: ISH visualizing *nanos2* expression throughout development;

B: *nanos2*::mOr expression; C: ISH visualizing *piwi1* expression throughout development; D: *piwi1*::mOr expression; E-F: *nanos2* and *piwi1* reporter gene constructs.

Fig. S2

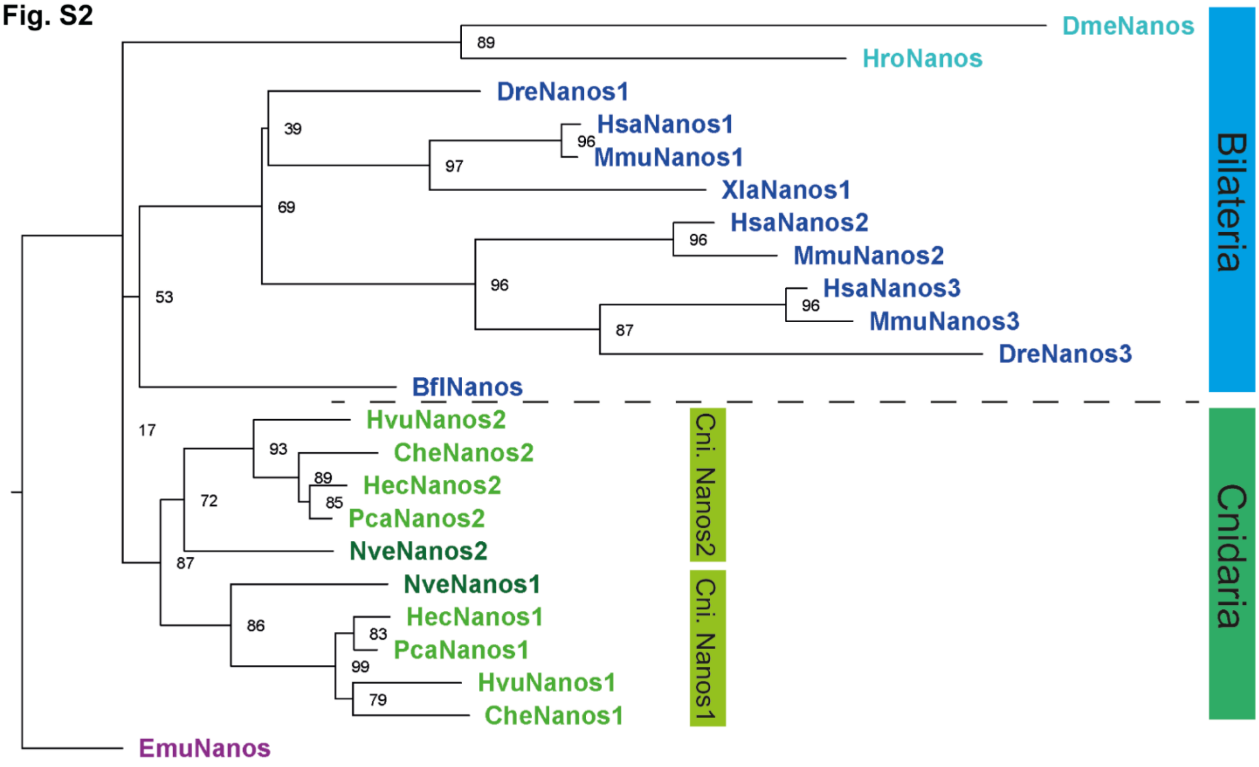

S2 Fig: Phylogenetic analysis of Nanos proteins. Bayesian consensus tree of metazoan Nanos proteins; Numbers at the branches indicate bootstrap support (1000 iterations). *Nanos* genes have duplicated in the cnidarian lineage and triplicated at the base of the teleost lineage independently. Dme: *Drosophila melanogaster*; Hro: *Hirundo robusta*; Dre: *Danio rerio*; Hsa: *Homo sapiens*; Mmu: *Mus musculus*; Xla: *Xenopus laevis*; Bfl: *Branchiostoma floridae*; Hvu: *Hydra vulgaris*; Che: *Clytia hemisphaerica*; Hec: *Hydractinia echinata*; Pca: *Podocoryne carnea*; Nve: *Nematostella vectensis*; Emu: *Ephydatia muelleri*.

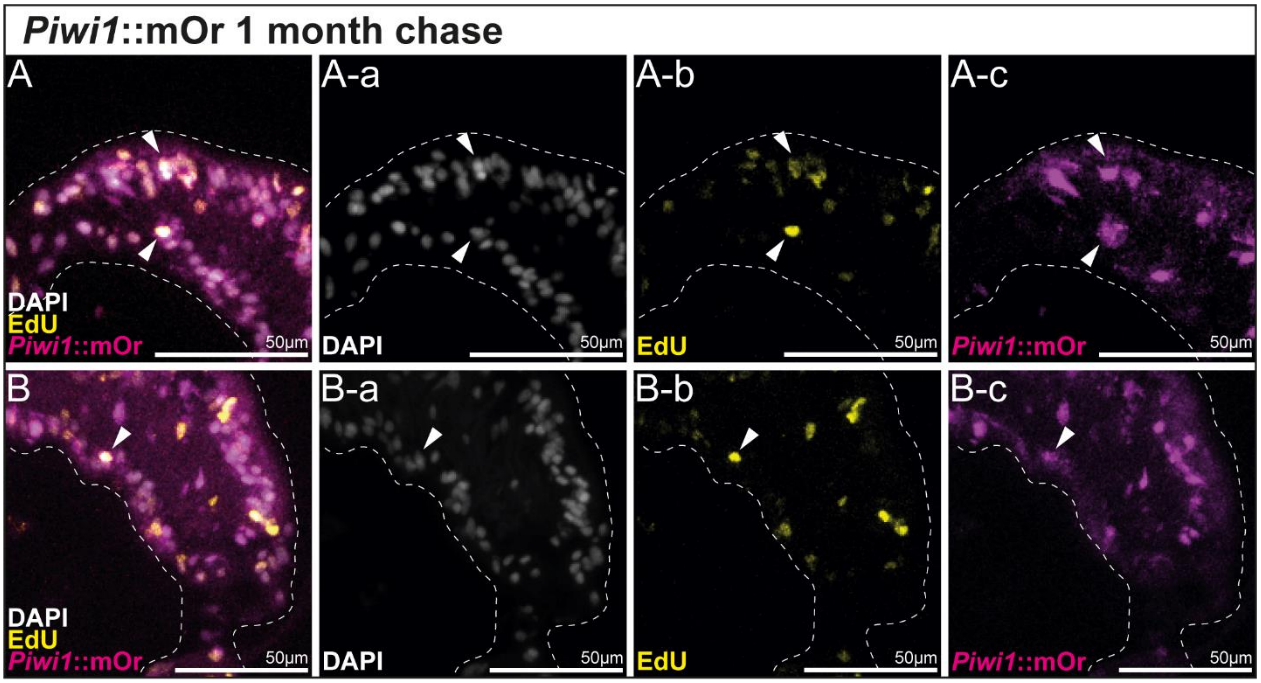

S3 Fig: Long term EdU label-retention assay in *piwi1*::mOr transgenic animals. A-C: EdU signal is retained in few mOr+ cells in the mesentery epithelia over 1 month, marking a subpopulation of slowly cycling cells. A, B: 1 month label retention in mesenteries. A-a, B-a: DAPI only. A-b, B-b: EdU only. A-c, B-c: mOrange only.

Fig. S4

```
ATGTCTTTGAGTGAGGTGATGTTAAAAACGCTGATTCCCAGCAACTTTTTGGACTCAGACGACGAAGAGATAAAG
TCGTTAACGTGCACTACGGAGAGCGATGATTTGCAGAAAAATCAGTATACCAACTACAACATGTTCAACGACTAC
TTCGGGCTCAGTAATCTGGTGCAAAAAGTGCGGCTCGCTAATAATGATTTCCCGTATCTCGGCAGCGATGGCTAC
GGCGGCAAACTGCACCGTCGTGACCGATTTGACAGCATTGATTCGGAGAACTCGTCTTCTGGAAGCTGTGATTCC
TTCACGGATCACGGCCTGACATTCGGCGAGGGATCATTTCAGCCATTTAGCACATCATCTATCCTGCACAGTGCA
GGGAAAAGGAAAGAAGTTGCGTTGCCGCCAAGTCGAAGGCTCGGAAAGCCGACCGCGCGAAGCTCAGCTCCCGGT
GCAAATCGTCAAGTGTGCGTGTTCTGCAGAAACAACGGTGAGAGCGGGAGGAGGTGTACGCTTCACATGTGTTAAA
AAGTGCCGATGGGAAAACTACTTGCCCGATCCTGAGAGCCTACACTTGTCCGATTTGCAAGGCAAGCGGCGATGA
TTCGCATACGATAAAATATTGCCCGCAAAATCAGCAAACGCAAGGAAATGGCCAGCTTCCTCCTCCACCAGTCAA
ACCACCAACCAGCACAGCACAGCCAATCGCTCGCTCCACGCGGGGAATAATGCCGCCAAAACGCCGCTAGACTAA
```

S4 Fig: *nanos2* genomic coding sequence of *nanos2* KO mutants. Nanos Zinc-finger sequence is highlighted in turquoise, 1bp insertion is highlighted in red.

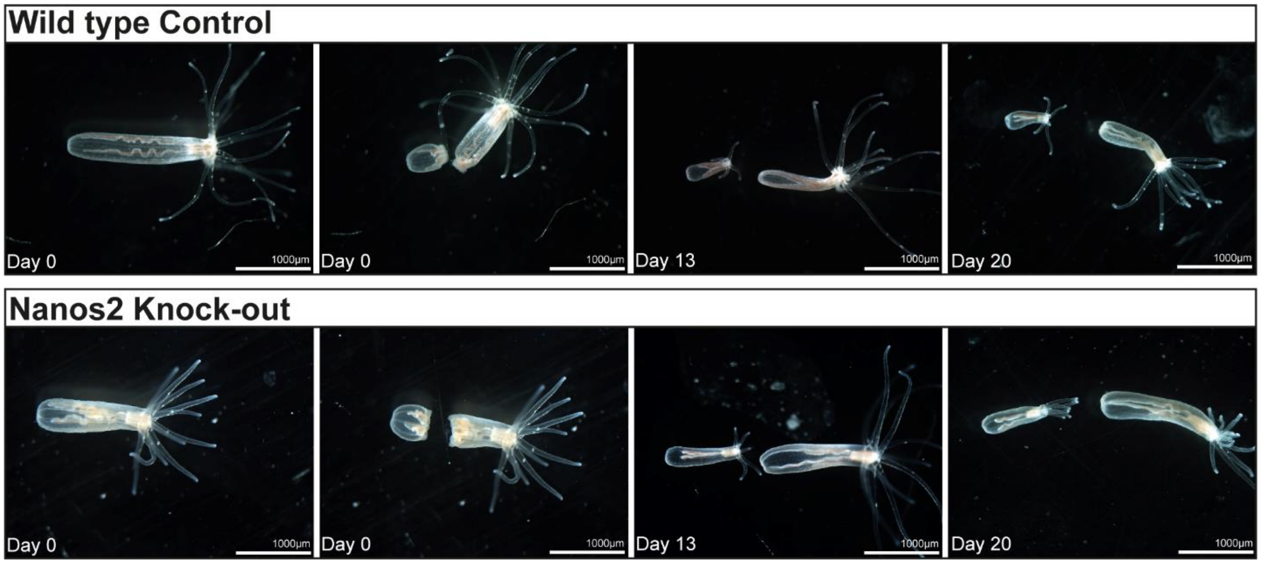

S5 Fig: Regeneration of Wild type (top row) and *nanos2* KO animals (bottom row). When regenerating from a mid-body bisection, *nanos2* KO animals regenerate in a similar fashion and speed to WT animals, resulting in wound closure and the reformation of a functional head (n=3).

Table 1: EdU counts of dissociated *nanos2* and *piwi1* Transgenic tissue

Table 2: Primers

Table 3: List of differentially expressed genes
